# Treat-to-target weight management in fibromyalgia: a preliminary estimate of clinically meaningful weight loss

**DOI:** 10.1093/rheumatology/keag611

**Published:** 2026-10-29

**Authors:** Jacopo Ciaffi, Jacob N Ablin, Valerie Aloush, Roberto Caporali, Francesco Ursini

**Affiliations:** Department of Biomedical and Neuromotor Sciences (DIBINEM), Alma Mater Studiorum University of Bologna, Bologna, Italy; Medicine and Rheumatology Unit, IRCCS Istituto Ortopedico Rizzoli, Bologna, Italy; Internal Medicine H, Tel Aviv Sourasky Medical Center, Tel Aviv, Israel; Gray Faculty of Medical and Health Sciences, Tel Aviv University, Tel Aviv, Israel; Gray Faculty of Medical and Health Sciences, Tel Aviv University, Tel Aviv, Israel; Internal Medicine A, Tel Aviv Sourasky Medical Center, Tel Aviv, Israel; Department of Clinical Sciences and Community Health, University of Milan, Milan, Italy; Department of Rheumatology and Medical Sciences, ASST Pini-CTO, Milan, Italy; Department of Biomedical and Neuromotor Sciences (DIBINEM), Alma Mater Studiorum University of Bologna, Bologna, Italy; Medicine and Rheumatology Unit, IRCCS Istituto Ortopedico Rizzoli, Bologna, Italy

*Rheumatology* key message • Mean weight losses near 15% and 30% may correspond to moderate and substantial fibromyalgia improvement.

Dear Editor, Clinicians routinely advise patients with fibromyalgia and obesity to lose weight, expecting symptomatic benefit. Yet this recommendation remains nonspecific, as we lack a clear understanding of how much weight loss is required for a clinically relevant benefit. Rheumatology has embraced a treat-to-target philosophy, defining quantitative goals that guide management in rheumatoid and psoriatic arthritis and increasingly framing obesity itself as a modifiable target with defined thresholds—∼7–8% weight loss for initial clinically meaningful benefit in knee osteoarthritis [1]. Fibromyalgia is a notable exception: the EULAR recommendations make no mention of weight status or weight reduction [2], and no quantitative target exists. This gap is striking given that obesity is highly prevalent in fibromyalgia and associated with virtually every clinical domain of the disorder [3], and that GLP-1 receptor agonists have recently been associated with reduced pain and opioid use in fibromyalgia [4]. In this letter, we therefore sought to provide a preliminary and exploratory estimate, based on the available evidence and irrespective of the weight-loss strategy used, of the magnitude of weight loss associated with clinically meaningful symptom improvement.

Building on the search underpinning our previous systematic review on fibromyalgia and obesity [3], further updated to April 2026, we selected interventional studies enrolling fibromyalgia patients (ACR 1990 or 2010/2016 criteria) with overweight or obesity undergoing a weight-reducing intervention with intentional weight loss as a therapeutic aim; elimination diets without a primary weight-loss objective were excluded. Because studies used different instruments—pain intensity (visual analogue scale or Brief Pain Inventory), the multidomain Fibromyalgia Impact Questionnaire or the Widespread Pain Index—we harmonized them into a single metric, expressed as percentage improvement from baseline and averaging pain and Fibromyalgia Impact Questionnaire where both were reported. Percentage weight loss was taken at the timepoint of maximum benefit and derived from BMI where body weight was not reported. We defined a priori the >30% and >50% improvement levels as the thresholds of interest, mirroring the benchmarks for moderate and substantial clinically important pain reduction established by IMMPACT [5] and applied to fibromyalgia [6]. Studies were stratified a priori into a conventional group (hypocaloric diet and bariatric surgery) and a very low-energy or ketogenic diet group.

Because our objective was to explore whether progressively greater weight loss was associated with increasing symptom benefit, without assuming proportionality between the two, we selected a nonlinear Hill model allowing a non-proportional relationship between weight loss and symptom benefit. Each study contributed a single pair of aggregate estimates—mean percentage weight loss and mean percentage symptom improvement—so that the fitted curve represented a study-level rather than individual-patient relationship. The three-parameter model was fitted to the conventional group by weighted nonlinear least squares, with thresholds obtained by algebraic inversion and 95% bootstrap CIs (3000 replications). Analyses were performed using R version 4.5.1.

Eight studies (405 patients) were included [7–14]. Five conventional studies entered the Hill model. The fitted curve indicated a nonlinear, near-hyperbolic relationship (Hill coefficient 1.22), with progressively greater weight loss associated with greater symptom improvement, but without implying a constant increase in benefit for each additional percentage point of weight loss. Inverting the fitted curve, our two prespecified thresholds corresponded to 15.3% weight loss (95% CI 11.4–19.9) for >30% improvement and 30.6% weight loss (95% CI 25.4–50.0) for >50% improvement (Fig. 1); smaller gains of >14% and >20% were reached at 6.9% and 9.8% weight loss, respectively. As no study exceeded 40% weight loss, the upper portion of the curve was poorly constrained by the observed data, reinforcing the exploratory nature of these model-derived estimates. Leave-one-out re-estimation nevertheless showed limited variation in the >30% improvement threshold (12.3–16.7% weight loss). A single pooled model failed to converge, indicating that a common curve could not be reliably fitted across these mechanistically distinct interventions.

**Figure 1:**
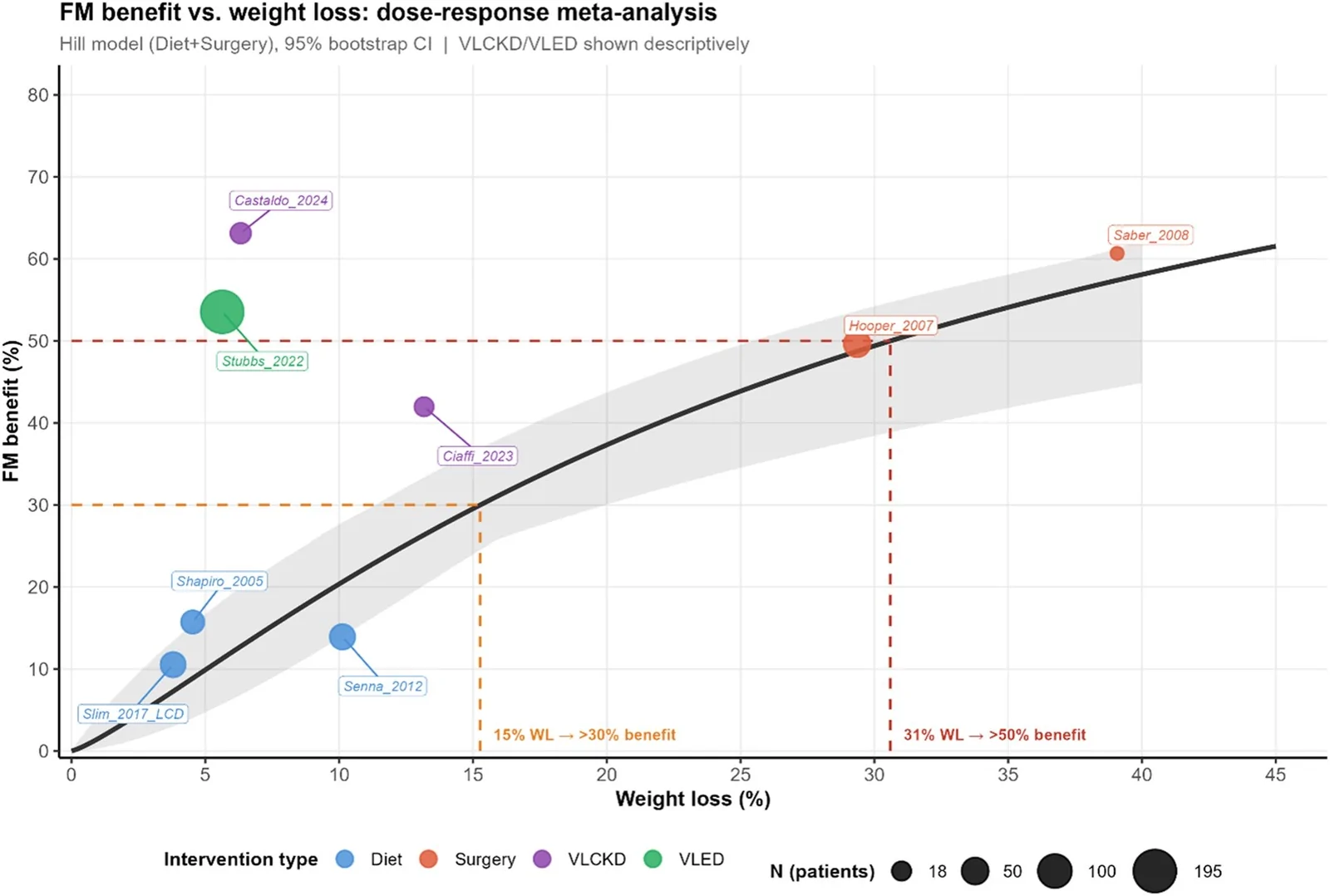
Relationship between weight loss and fibromyalgia symptom improvement. Each point represents one study, plotted by percentage weight loss and percentage fibromyalgia benefit, with point size proportional to sample size and colour denoting intervention type. The solid line shows the weighted three-parameter Hill model fitted to the conventional weight-loss studies (hypocaloric diet and bariatric surgery), with the shaded band representing the 95% bootstrap CI (3000 replications). Dashed lines indicate the weight loss associated with >30% fibromyalgia benefit (15.3% weight loss) and >50% fibromyalgia benefit (30.6% weight loss). The very low-energy/ketogenic diet studies are displayed for reference but were not included in the model. FM: fibromyalgia; VLED: very low-energy diet; VLCKD: very low-calorie ketogenic diet; WL: weight loss

Three very low-energy/ketogenic diet studies fell markedly above the conventional curve, achieving 42–63% benefit at only 6–13% weight loss [12–14]. This dissociation may reflect weight-independent effects of severe calorie restriction and ketosis—including modulation of central pain processing and neuroinflammation—that merit dedicated investigation [13].

In our model, the >50% symptom-improvement threshold corresponded to weight loss approaching 30%—a magnitude generally attainable only through bariatric surgery—whereas the >30% improvement threshold fell within the range achieved in the pivotal trials of semaglutide and tirzepatide [15]. This overlap is clinically relevant because weight loss of this magnitude, previously difficult to achieve without surgery, is now increasingly feasible with pharmacological treatment, providing a rationale for prospective testing of whether incretin-induced weight loss translates into clinically meaningful symptom improvement in patients with fibromyalgia and obesity.

These estimates should be read as exploratory and hypothesis-generating rather than as individual thresholds or established treat-to-target goals. They derive from a small number of heterogeneous studies and rest on a composite improvement metric, whereas the IMMPACT thresholds were validated for pain intensity alone. Further limitations include selection of the timepoint of maximum benefit, which combines heterogeneous follow-up durations, and the stability of a nonlinear model based on only five studies; hypocaloric diet and bariatric surgery, whose weight-independent effects may differ, could not be analysed separately. Importantly, the fitted relationship was nonlinear and should not be interpreted as evidence of a constant or directly proportional increase in symptom benefit with increasing weight loss. Moreover, these estimates describe a study-level association between mean weight loss and mean symptom improvement and should not be interpreted as individual-level dose-response thresholds. Thus, a mean weight loss of ∼15% associated with >30% improvement across studies does not imply that an individual patient losing 15% of body weight will necessarily experience a corresponding degree of symptom improvement. Nor can the available data establish whether weight loss itself mediates the observed benefit, since weight-reducing interventions may influence fibromyalgia symptoms through mechanisms partly independent of weight change.

Within these constraints, study-level data suggest that mean weight losses of ∼15% and 30% may be associated with moderate and substantial symptom improvement, offering a preliminary quantitative anchor to replace vague encouragement to ‘lose weight’ and a first step toward bringing weight into rheumatology’s treat-to-target framework.

## Data Availability

All data analysed derive from the published studies cited; extracted data are available from the corresponding author on reasonable request.
